# HIV and risk behaviors of persons of low socio-economic status, Popayan-Colombia (2008-2009)

**Published:** 2013-03-30

**Authors:** Hector Fabio Mueses, María Virgínia Pinzón, Ines Constanza Tello, Hernan Gilberto Rincón-Hoyos, Jaime Galindo

**Affiliations:** a Grupo educación y salud en VIH /Sida de la Corporación de Lucha Contra el Sida, E-mails: jaimegalindo@cls.org.co; b Universidad del Cauca, Colombia.

**Keywords:** HIV, risk factors, health promotion, alcohol drinking, adults, poverty

## Abstract

**Abstract:**

**Objetive:**

To determine HIV presence and risk behaviors of persons of low socio-economic status in the city of Popayan-Colombia.

**Methods:**

Cross-sectional study; between 2008 and 2009, 363 participants of Popayan signed informed consent and received pre and post HIV test counseling. Socio-demographic characteristics and history of STDs, risk behaviors and previous HIV testing were assessed. Descriptive statistics, correlations and multivariate logistic regression were calculated.

**Results:**

Mean age 33.5±10,2; 66 %women. Frequency of HIV-positive patients was 3.86 % (95% CI:1.87-5.85), greater in men (7.38%; *p*= 0.013). Greater frequency of HIV-positive patients was observed in people age 29-37, those without a stable partner, and those with history of risky alcohol consumption (more than five drinks in 2 h).

**Conclusions:**

HIV-positive patients frequency in this population was greater than national estimate for general population, aged 15-49 in Colombia, with even greater frequency in men. This study suggests that characteristics associated with low socioeconomic status, in economically active population, without a stable partner and with risky alcohol use, can potentially increase risk of HIV infection.

## Abstract (es)

**Objetivos:**

Determinar la frecuencia de VIH y comportamientos de personas en contexto de vulnerabilidad por estrato socioeconómico bajo de la ciudad de Popayán-Colombia.

**Métodos:**

Estudio transversal; entre 2008 y 2009, 363 participantes de Popayán dieron su consentimiento informado y recibieron consejería previa y posterior a la prueba del VIH. Se evaluaron características socio-demográficas, antecedentes de enfermedades de transmisión sexual, comportamientos y prueba previa del VIH. Se calcularon estadísticas descriptivas, correlaciones y regresión logística multivariada.

**Resultados:**

La edad promedio 33.5 ± 10.2; 66% mujeres. La frecuencia de pacientes VIH positivo fue 3.86% (IC95: 1.87-5 .85), mayor en hombres (7.38%, *p*= 0.013). Personas de 29-37 años de edad, sin una pareja estable y aquellos con historia de consumir más de cinco copas de licor en dos horas seguidas presentaron mayor frecuencia de pacientes VIH positivo.

**Conclusiones:**

La positividad para VIH en esta población es mayor que el estimado nacional para población general en edad 15-49, con frecuencia aún mayor en hombres. Los datos sugieren que la vulnerabilidad asociada con un estrato socioeconómico bajo en la población económicamente activa, sin una pareja estable y con consumo riesgoso de alcohol, puede aumentar potencialmente el riesgo de infección por el VIH.

## Introduction

In spite of the important advances made through prevention programmes to avoid new infection and deaths, HIV/AIDS remains as one of the main priorities in global public health, given that epidemic is still on the rise^1^.

In Colombia, according to the National Social Protection Agency HIV observatory, since 1983 the cumulative total cases reported as of 2008 were 64,729. Therefore, the prevalence remains below 1.2%, concentrated in homosexual and bisexual men^2^. With respect to specific subpopulations, Colombian estimates show that the most-at-risk groups for HIV infection in the country are: a) men who have sex with men (MSM), b) sex workers, c) injecting drugs users (IDU), d) teenagers, e) pregnant women, f) population deprived of liberty and g) forcibly displaced population.

An aspect that has been shown to be directly correlated to population's vulnerability to HIV is low income level; apparently, the reason for this is that people under this condition are more prone to engage in sexual transactions or intercourse under circumstances that hinder the adoption of protective behaviors^3^. This could be an explanation for the fact that epidemic ends up moving towards the poorer demographic groups, no matter the income level of the country^4^. The higher prevalence rates of sexually transmitted diseases (STD) among lowest income groups^5^ ^,^ ^6^, seems to corroborate the stated hypothesis.

Furthermore, reports indicate many HIV infected people do not take the test until the infection is in an advanced stage^7^ ^,^ ^8^. In addition, access to diagnose is hampered by a variety of factors such as: stigma, fear of discrimination^9^ and cost of the test. In this regard, it is important to point out that is frequent to find lack of access to testing, especially among lowest income people, due to limited or ineffective prevention plans and services targeted at them.

Popayan is the main city of the Cauca department, and according to the Colombian national census data, it has a population of 258,653 people. From these, 64.23% are registered in the System for selection of potential beneficiaries to social programs (SISBEN is the acronym in Spanish), and belong to the lowest socioeconomic strata (1 and 2) (Office of Popayan's Mayor, Municipality Planning Agency, September 18th, 2008).

In relation to HIV epidemic status, for 2008 the prevalence for Popayan was estimated at 0.1%; however, there are currently no studies that disclose the present magnitude of the problem, neither the demographic features of the affected population.

In addition, the proper identification of behavioral risk factors of some city's specific populations may be useful to guide and optimize the resources invested in developing strategies for promotion and prevention. Considering this, a study was carried out with the purpose of determining the frequency of HIV, as well as identifying behaviors of asymptomatic, low income people in the city of Popayan, Colombia, between years 2008 and 2009.

Besides the generation of epidemiologic knowledge for decision-making, it is expected that results of this study will contribute to improve care of HIV positive people in Popayan, including early detection, health services reorientation and access to treatment.

## Materials and Methods

A cross sectional study was carried out as part of the Strategy of Active, Focused Comprehensive Search Program (BAFI is the acronym in Spanish) implemented by the Corporacion de Lucha Contra el Sida in the city of Popayan. During years 2008 and 2009, using a sampling by convenience, 363 people were voluntarily interviewed, all of them adults from lowest socioeconomic strata (1-2)

Study participants were recruited by community leaders from low income neighborhoods, social organizations and municipality authorities. Study activities were done at community associations' centers. Participating neighborhoods were those with lowest income in the city, which harbor people living in poor, unhealthy dwelling conditions that are characteristic of these communities^10^: a) inadequate walls, floors or ceilings; b) lack of at least one public utility, c) overcrowding (3 or more people per room), d) high dependency, i.e. more than three people depending on a low-educated (two or less years of schooling) head of household and e) in each home, at least one of the children in school age is not attending school.

Adults without previous diagnose of HIV were included in the study. Every participant signed an informed consent form, received pre and post-test counseling, and a blood sample HIV screening test was provided.

A face to face interview was carried out with each participant and a structured questionnaire (SQ) was applied. This questionnaire included social and demographic characteristics, sexual practices and habits. The SQ was applied prior to counseling and assessment sessions in order to prevent possible biased answers.

The interviews were conducted by specifically trained health professionals, and the SQ was addressed in a physical environment that fulfilled conditions to warrant participant's right to privacy. To fill out the questionnaire took approximately 15 min.

The qualitative immunoassay DetermineTM HIV-1/2 for detection of antibodies to HIV was used as screening test.

A trained professional bacteriologist collected and processed the samples. In case the first screening test was positive, a second presumptive test was to be performed. Given the case it was also positive; a Western-Blot assay was performed for confirmation. Participants with confirmed HIV diagnosis were referred to clinical assessment and to an HIV care program, based on participant's health insurance coverage. HIV negative participants were educated in a post-test counseling session that took place after delivery of results.

### Ethical aspects

Participation in the study was completely voluntary, and a properly informed consent form was obtained from each participant, both for HIV testing and the SQ and interview as well. 

This study was reviewed and approved by the Institutional Review Board of the Corporacion de Lucha Contra el Sida;, and following Colombian resolution number 8,430 of 1993, article 11, title 2; the study was classified in the minimum risk category.

### Statistical analysis

A database using EpiInfo(r) Version 3.41 was created, and statistical packages SPSS(r) version 17 and STATA(r) version 9 were employed for data analysis. To ensure the quality of the information, database review and cleaning was performed.

For each variable measured, an exploratory data analysis was carried out calculating central tendency measures, dispersion, frequency tables and 95% confidence intervals.

The possibility of recoding the numerical variable "age" was explored in order to determine which categories exhibited relationships with Odds Ratio greater than "1", and to simplify univariate and multivariate analysis; different cut off points were explored, categorization in tertiles, quartiles and quintiles was examined, and ORs were plotted for each categorization. The most appropriate cut off points were identified and recoding of the variable was performed.

Raw relationships were estimated between covariates and the dependent variable "HIV diagnosis". Odds ratios and 95% confidence intervals were calculated. In accordance with Hosmer and Lemeshow, those variables with p-values less than 0.25 were included in a multivariate logistic regression model that allowed identifying variables associated with HIV positive diagnosis.

## Results

### General characteristics:

As part of the BAFI search strategy between the years 2008 and 2009; 363 adults from the socioeconomic strata 1 and 2, living currently in Popayan, were interviewed. The mean age of the participants was 33.5 ± 10.2 years. The main demographic results are shown in Table 1.

**Table 1:**
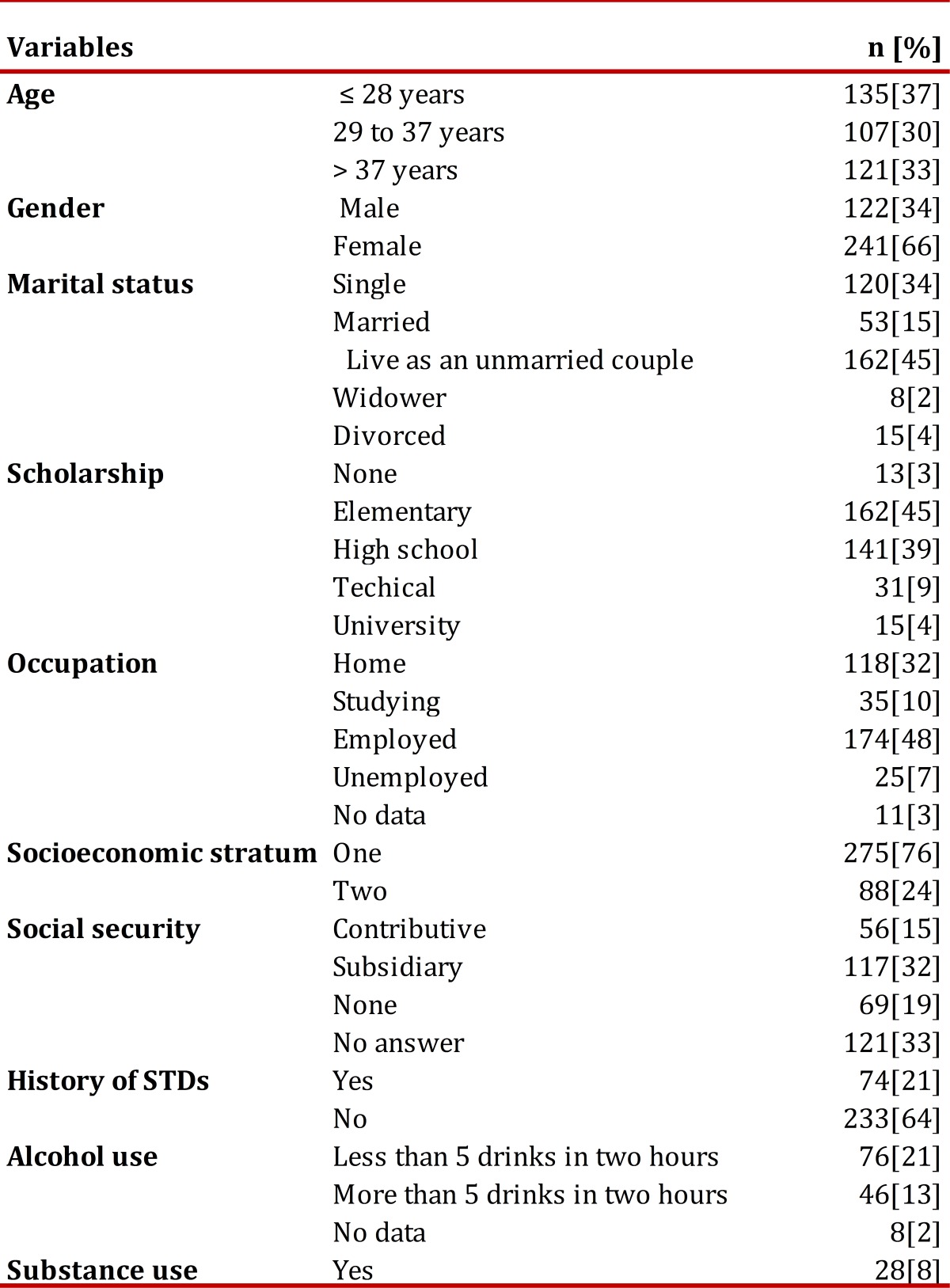
General characteristics of participants. Active, Focused Comprehensive Search Popayán, 2008-2009.

### Background of participants:

Most participants (89%) reported no history of blood transfusion; about one fifth of the sample (22%) said they had a piercing or tattoo done, and 21% reported a history of sexually transmitted diseases (STDs) ( Table 1).

### Substance use:

Thirty four percent of participants (122/363) reported current use of alcohol, and 38% of those said they drank more than five (5) drinks or bottles of beer in less than two hours. A relative low percentage (8%) reported other drugs use, which were mainly marijuana, powdered cocaine (perico) and paste cocaine (basuco).

### Sexual practices:

The average age of first sexual intercourse was 16.2 years, with a standard error of 0.18, and was lower in males than in females (15.3 yrs versus 16.7 yrs; *p*= 0.0002). When asked about the number of sexual partners in the last year, most participants (71%) reported a single one, while 26 percent reported between two (2) and five (5) partners. 

Six percent reported consistent (always) use of condom; 13% reported practicing anal sexual intercourse, being more frequent in males than in females (19% vs. 9.5 %; *p*= 0.027). Twenty one percent of participants had engaged in sexual intercourse during women's menstruation period.

### HIV prevalence: 

The sample's overall prevalence of confirmed HIV positive was 3.86% (1.87-5.85; 95% CI), being higher in males (7.38 % versus 2.07%; *p*= 0.013)

### Results related to HIV diagnosis and study covariates:

Results from the bivariate analysis were significant by sex and occupation. Males and unemployed subjects had a higher probability of a confirmed HIV positive diagnosis (Table 2).

**Table 2.**
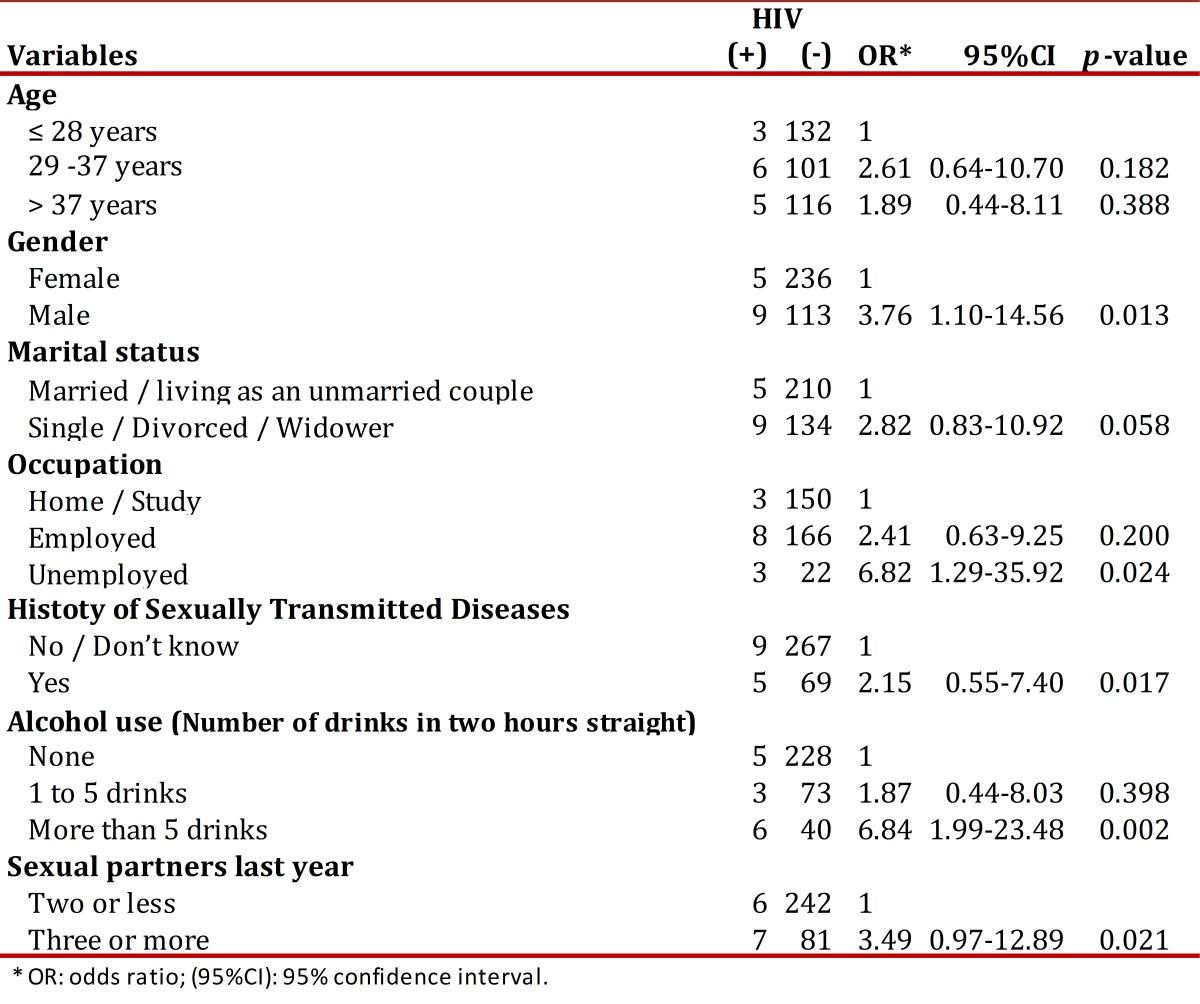
Bivariate analysis of the HIV test result, associated with demographic and behavioral features, with significance less than 0.25. Active, Focused Comprehensive Search Popayan, 2008-2009.

A positive relationship between having three or more sexual partners during last year and being HIV positive diagnosis was found ( Table 2). The percentage of HIV positive was 2.4%, 3.46% and 14.3% among those reporting one (73%), two, or three-plus partners in the last year respectively.

In addition, HIV positive rates were increasingly higher in subjects not living with a stable partner. In this group there were significant differences in prevalence associated with number of sexual partners during last year (one or no sexual partner: 3.9%, two partners: 3.0% and three or more partners 25%) when compared to those living with a stable partner. The latter group showed no prevalence differences in the split analysis related to number of sexual partners last year (one or no sexual partner: 1.8%, two partners: 4.6% and three or more partners 5.6%).

No differences in HIV positive rates were found to be associated with the following variables: age, scholarship, time in sexually active life; history of blood transfusion, tattoos or piercings, substance abuse, consistent use of condom, and practice of anal sex or intercourse during menses.

### Multivariate logistic regression model results:

The variables reported in Table 2. were looked at in a multivariate model analysis showing a higher probability of HIV positive diagnosis in people in the 29-37 years age interval group, those not living with a stable partner, as well as in those with history of five or more drinks in less than two hours. The model obtained is shown in Table 3.

**Table 3:**
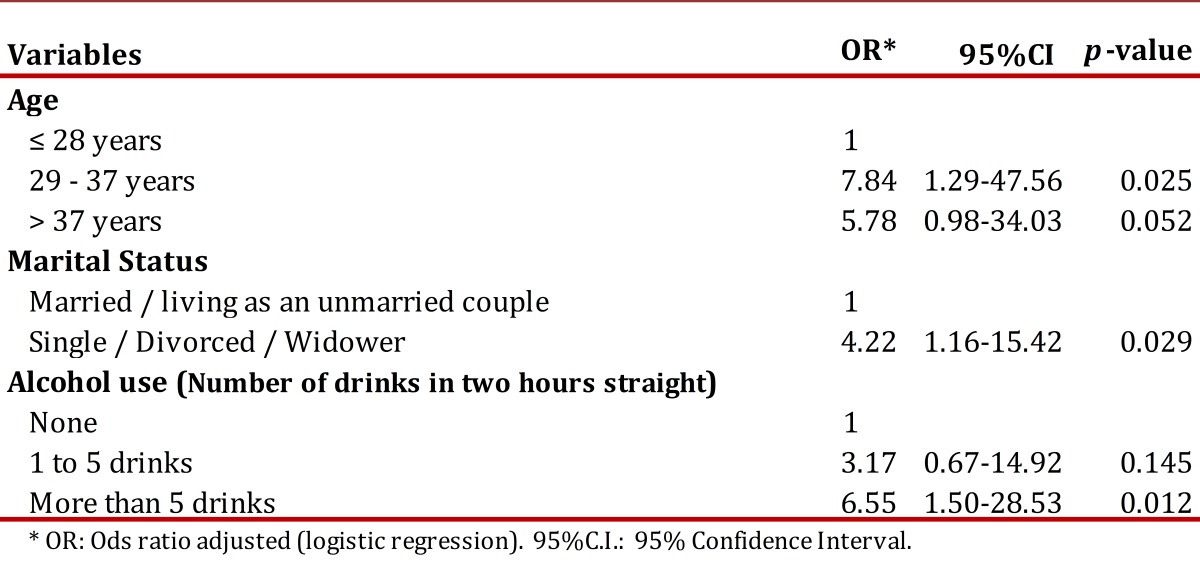
Variables related to HIV positive diagnosis. Active, Focused Comprehensive Search Popayán, 2008-2009.

## Discussion

The results of this study are important to understand behavioral factors relevant to HIV infection phenomenon in the city of Popayan; and consequently as an input for designing and planning intervention strategies to counter these behaviors, perhaps contributing to diminish the threat posed by dissemination of the HIV/AIDS epidemic.

In the studied population, the overall frequency of HIV-Positive cases (3.86%) found was higher than estimated (0.7%) for general Colombian population in the 15-49 age interval and also than results shown elsewhere under the BAFI search strategy^11^. Although male participation was lower than female participation, they had higher probability of HIV infection, which is consistent with prevalence distribution in the country^12^. However, an in-depth inquiry about sexual preferences was not performed, which could possibly allow a better understanding of characteristics of HIV-positive males.

Even though the sub-population under study may have higher risk, these findings suggest a notorious increment in the HIV frequency rates related to low socioeconomic strata, mainly among male participants. Therefore, intervention strategies for preventing HIV and other STDs must likely be differentiated based on gender and other factors.

The presented results suggest that the most frequent age-at-diagnosis was between 29 and 37 yrs, which according to natural course of disease, most likely acquired the infection two to five yrs before. Therefore, health promotion strategies and primary prevention interventions should perhaps be directed towards the adolescent population in the 15-24 yrs range, and be complemented by secondary and tertiary health promotion interventions targeted towards the 25 years and older group.

It was also found that those who reported not living with a stable partner had higher frequency rates of HIV infection. In this regard, it has been documented that sexual risk behaviors are heterogeneous, and that HIV transmission risk among people in stable relationships varies according to environment. Nevertheless, literature review suggests that the HIV infection risks increases with the number of sexual partners^13^ ^,^ ^14^. In this study, the descriptive analysis yielded a relationship between the number of partners and HIV positive diagnosis, but this relationship disappeared when adjusted for other covariates in the multivariate model.

Binge drinking, i.e. taking more than 5 drinks of an alcoholic beverage in less than two hours, was found to be correlated with higher probability of HIV positive diagnosis. This relationship is explained due to the negative effects of alcohol use on cognitive functioning, such as judgment impairment, which can hinder the person's ability to avoid engaging in HIV and STDs risks behaviors.

In this regard, it has been acknowledged that binge drinking may lead to behaviors that facilitate HIV infection^15^ ^,^ ^16^. For example, an intoxicated person would have difficulty in refusing to engage in a risky sexual behavior, and would have neither interest nor ability to use a condom properly. Furthermore, among people living with HIV, excessive alcohol use has been associated with increase of medical and psychiatric complications, delaying treatment^17^ and adherence problems^18^ ^,^ ^19^. All of the above are harmful situations that not only put themselves at risk of further infections, but also others due to increased likelihood of transmission.

Among other relevant findings, this study has identified a relationship between history of sexually transmitted diseases and HIV diagnosis, which has been widely described^20^ ^-^ ^21^. This highlights the importance of STDs prevention and control as a lead tool for HIV screening.

Based on this study, the governments of the City of Popayan and Cauca Department have the possibility of starting dual and partner strategies oriented towards prevention of both HIV risk behaviors including alcohol abuse. These strategies should be targeted at the lowest income population, and should be differentiated by gender and adjusted for older age groups.

As has been suggested above, these findings compel to further inquire in the relation of alcohol use and HIV diagnosis; which may be addressed in the future with alternative and more appropriate methodologies both to explore the relationship, and to understand the behavioral and contextual factors leading to increase of HIV infection risk related to alcohol use. Of the 14 confirmed HIV positive cases obtained among the sample, 13 attended their first medical visit. The other participant was not located, therefore was declared as a loss to follow up. Despite the unfavorable demographic conditions of the participants, a notably high response rate to follow up (13/14, 93% of positive cases) was achieved, possibly due to efforts of the health care personnel involved. In other similar studies, only between 45% and 79% of the subjects returned to receive post-test counseling^12^ ^,^ ^22^. Likewise, data from Centers for Disease Control and Prevention (CDC) indicate that about one third of the people who take the HIV test never return to get their results^23^.

It is important to highlight that referral to an HIV clinical program was accepted and complied with by 93% (13/14) of the HIV positive participants. Of these, 7 were found to require and were put on antiretroviral therapy, 5 did not meet the clinical criteria to initiate treatment, and the remaining one did need to initiate treatment, but was non-adherent due to drug addiction issues.

To sum up the study findings, due to unfavorable social and environmental conditions, HIV prevalence is likely to remain on the rise among the most socioeconomically vulnerable population Adolescence, young adulthood, binge drinking, and absence of stable partner are likely to be important risk factors in these demographic groups. Other factors to take into consideration are male gender and number of sexual partners, especially among who do not live with a stable partner.

Regarding the study limitations, even though a high frequency rate of HIV positive cases was found, this could not be extrapolated to population-level data, given that probabilistic sampling was not carried out. Additionally, participants were a self-selecting sample, probably on the basis of their own perception of risk, knowledge, and beliefs of stigma and discrimination related to HIV disease.
